# Self-Management and Its Associated Factors Among People Living With HIV at University of Gondar Comprehensive Specialized Hospital: A Cross-Sectional Study

**DOI:** 10.1155/2024/5590331

**Published:** 2024-11-05

**Authors:** Abdisa Gemedi Jara, Masho Tigabe Tekle, Faisel Dula Sema, Banchamlak Teferi Mekonen, Asrat Elias Ergena, Amensisa Hailu Tesfaye, Saron Naji Gebremariam, Rahel Belete Abebe, Eyayaw Ashete Belachew, Abenezer Melaku Tafese, Eden Abetu Mehari

**Affiliations:** ^1^Department of Clinical Pharmacy, School of Pharmacy, College of Medicine and Health Sciences, University of Gondar, Gondar, Ethiopia; ^2^Department of Pharmaceutical Chemistry, School of Pharmacy, College of Medicine and Health Sciences, University of Gondar, Gondar, Ethiopia; ^3^Department of Environmental and Occupational Health and Safety, Institute of Public Health, College of Medicine and Health Sciences, University of Gondar, Gondar, Ethiopia; ^4^Department of Internal Medicine, School of Medicine, College of Medicine and Health Sciences, University of Gondar, Gondar, Ethiopia

**Keywords:** HIV/AIDS, people living with HIV, process of self-management, self-management

## Abstract

**Background:** Self-management (SM) is the gold standard of care and is the direct active participation of patients in their disease management. Condition-specific factors, physical and social environment, individual and family, and the process of SM are factors that influence SM in people living with human immunodeficiency virus (PLHIV). Poor SM leads to high retroviral infection transmission, mortality, and morbidity.

**Objective:** This study was aimed at assessing SM and its associated factors among PLHIV at the University of Gondar Comprehensive Specialized Hospital (UOGCSH), Northwest Ethiopia.

**Methods:** A cross-sectional study was conducted using a systematic random sampling technique at the UOGCSH from May 20 to July 30, 2022. The data were collected using a previously validated tool and were entered and analyzed using Statistical Package for Social Sciences Version 25. A binary logistic regression analysis was used to identify factors associated with poor SM. The statistical significance was considered at a *p* value < 0.05.

**Result:** Of 419 PLHIV, the median (IQR) SM score was 39 (9), and above half (52.6%, 95% CI: 48%–57%) of them had poor SM. Being unemployed (AOR = 2.49, 95%CI = 1.19, 5.19), living alone (AOR = 2.16, 95%CI = 1.12, 4.17), unfamiliar with the management of HIV-related symptoms (AOR = 3.59, 95%CI = 2.08, 6.20), poor social support (AOR = 3.02, 95%CI = 1.54, 5.93), poor self-efficacy (AOR = 3.04, 95%CI = 1.83, 5.06), and unsupported by the adherence support group (AOR = 17.17, 95%CI = 8.37, 35.22) were significantly associated with poor SM.

**Conclusion:** The majority of PLHIV had poor SM. This study supports the findings of individual family SM theory and previously published studies regarding factors affecting SM. The government, hospital, adherence support groups, and PLHIV should work on modifiable sociodemographic, condition-specific, and process of SM to improve SM of PLHIV.

## 1. Background

Globally, 84.2 million people become infected with the human immunodeficiency virus (HIV), and 40.1 million people have died from acquired immune deficiency syndrome (AIDS)–related illnesses since the start of the epidemic. In 2021, 38.4 million people were living with HIV, and 1.5 million people became newly infected. In 2020, HIV/AIDS cost the world around 21.5 billion, $5 times higher than in 2000 [1].

The development and widespread availability of antiretroviral treatment (ART) has transformed HIV into a chronic illness [2], which requires continuous self-management (SM) [3, 4]. The idea of SM was first stated by Thomas Creer when he wrote about the rehabilitation of chronically ill asthmatic patients 40 years back [5]. SM is the gold standard of care in chronic diseases [6]. It is patients' direct active participation in their disease management and contains disease management, lifestyle modification, and dealing with the chronicity of diseases [7]. SM improves individual health and quality of life and decreases the progression of the disease. Moreover, it has a crucial role in preventing communicable and noncommunicable diseases [8, 9].

Individual family self-management theory (IFSMT) states that SM can be influenced by condition-specific factors, the physical and social environment, individual and family factors, and the process of SM. The process of SM includes knowledge and beliefs about HIV, enhancement of self-regulation abilities, and social facilitation. In people living with HIV (PLHIV), SM is a lifelong task that requires better practice than yesterday; it may be substantially affected by the social contexts in which they live [10]. In addition to increased overall healthcare-related costs, hospitalization, emergency room visits, mortality, and morbidity [11–13], poor SM decreases social activities and functions, communication with physicians, and self-efficacy, and it worsens HIV/AIDS progression and physical comorbidity. These lead to a sedentary lifestyle, anxiety, depression, the practice of unsafe sexual intercourse, and increased HIV transmission [14–19]. Furthermore, it negatively affects medication adherence [18], which in turn increases the viral load, decreases the CD4 level, and increases disease progression [20]. Finally, poor SM leads to failed ART programs and poor quality of life and increases the burden of disease [21, 22].

Studies in many countries reported that PLHIV has a poor SM [23–26]. However, there is limited evidence regarding the SM of PLHIV in low- and middle-income countries. In Ethiopia, despite increased intervention and resource mobilization, the burden of HIV/AIDS remains high [27]. In 2021, 513,863 people were living with HIV, 12,000 new infections, and 12,000 deaths of PLHIV were reported.

As far as the investigators' best literature review encompassed, there is only one single center study about SM of PLHIV in the Debre Markos Referral Hospital. However, there is a lack of evidence after the implementation of a dolutegravir-based ART regimen, which required different SM due to various side effects than the efavirenz-based regimen, and the impact of COVID-19 on the world, which affects the SM of people with chronic disease [28, 29]. Thus, this study was aimed at assessing SM and its associated factors among PLHIV attending the ART clinic at the University of Gondar Comprehensive Specialized Hospital (UOGCSH), Northwest Ethiopia. This study may help to show the SM level of PLHIV after the implementation of a dolutegravir-based ART regimen in the study setting and help to attract the attention of all interested stakeholders to improve the SM of PLHIV.

## 2. Materials and Methods

### 2.1. Study Setting and Design

A cross-sectional study was conducted at UOGCSH from May 20 to July 30, 2022. UOGCSH is found in Gondar town, 738 km from Addis Ababa (the capital city of Ethiopia), in Northwest Ethiopia. Currently, it is one of the top university hospitals in Ethiopia. It serves as a referral hospital for more than 7 million urban and rural inhabitants. It has around 1000 beds for inpatient services. It also has 14 units that provide outpatient services for around 250,000 patients per year. From these, a total of 5254 registered PLHIV were on follow-up at the ART clinic in 2021/22.

### 2.2. Study Population and Sampling

All PLHIV who had regular follow-ups at the UOGCSH ART clinic were the source population. The study population consisted of all PLHIV who had regular follow-ups and visited the UOGCSH ART clinic during the study period. PLHIV willing to participate, age greater than 18, and on ART for at least 6 months were included in this study. Psychiatric PLHIV, PLHIV who cannot care for themselves (bedridden), severely ill PLHIV who visited the ART clinic medical unit, and PLHIV with difficulty communicating (any listening and talking problems) were excluded from this study.

The sample size was determined using a single population mean formula. The standard deviation of poor SM (0.22) and the precision of the study (0.0196) were taken from a previous study [23]. Finally, after adding a 15% contingency for the nonresponse rate, the total sample size of this study was 514.

 $$\begin{array}{c} N={\left(z\alpha /2\right)}^{2}S^{2}/{\left(\text{precision}\right)}^{2},\text{Precision}=S/\sqrt{n}\times \left(z\alpha /2\right),\text{Precision}=0.22/\left(\surd 415\right)\times \text{z}\alpha /2=0.0196,N={\left(1.96\right)}^{2}0.22^{2}/{\left(0.0196\right)}^{2}=489. \end{array}$$

Because the PLHIV was 5254, nf = *no*/(1 + *no*/*N*) = 489/(1 + 489/5254) = 447.

nf with (contingency 15%) = (447 × 0.15) + 447 = 514where *N* is the sample size, *S* is the standard deviation, *n* is the sample size from a previous study, *zα*/2 is the confidence interval at the 95% confidence level, and nf is the total sample size.

### 2.3. Data Collection Tool

The SM was measured by the 20-item SM scale that was developed by Webel et al. [30]. This SM scale contains three domains: daily SM health practices (12 items), social support (three items), and living with chronicity of HIV (five items). Each item was scored by a 4-point Likert scale ranging from 0 to 3 (0 = *not applicable*, 1 = *none of the time*, 2 = *some of the time*, and 3 = *all the time*). The total score of the scale was calculated by adding together all the items' scores. Possible scores range from 0 to 60. Its reliability statistical test (Cronbach's alpha) was 0.753 in this study. Permission to use this tool was obtained through email. PLHIV who scored overall SM above the median score were considered to have good SM, while the rest were considered PLHIV with poor SM.

Self-efficacy was measured by using an existing eight-item perceived medical condition SM scale applied to PLHIV. Overall, this item scored on a 3-point Likert scale (1 = *disagree*, 2 = *neutral*, and 3 = *agree*). The total score of self-efficacy was calculated by adding together all the item scores. Possible scores range from 8 to 24. A reverse scoring technique was used for Question Numbers 211, 212, 216, and 217 [31]. The reliability statistical test (Cronbach's alpha) was 0.877 in this study, and another study also used a similar data collection tool in this setting [2]. PLHIV who scored above the median value were considered to have good self-efficacy, and the rest were considered PLHIV with poor self-efficacy in this study.

Three individual yes-or-no questions developed by Areri, Marshall, and Harvey were used to assess the self-regulation ability and intervention of SM [23].

The social support of PLHIV was measured using the Oslo-3 Social Support Scale (OSS-3). The result was described as strong social support [12–14], intermediate social support [9–11], and poor social support [3–8, 32]. The reliability statistical test (Cronbach's alpha) for the social support tool was 0.667 in this study.

Adherence status was assessed based on the reported number of pills that had been missed 1 month before the data collection period divided by the number of prescribed pills multiplied by 100%. PLHIV who reported missed doses of > 5% of the prescribed medication were considered nonadherent [33].

### 2.4. Data Collection Procedure

The questionnaire was prepared in English, then translated into Amharic, and back-translated to English to minimize translation errors. Interviewer-administered questionnaires were used to collect the data by two clinical pharmacists after they received intensive training on the purpose of the study, methodology, data collection method, the confidentiality of information, participants' rights, and ethical aspects. Supervision of data collection was conducted by a senior clinical pharmacist on a daily basis. The supervisor and principal investigator reviewed and checked the consistency, completeness, and accuracy of data regularly.

The questionnaire was pretested on 50 PLHIV at the UOGCSH ART clinic 2 weeks before the actual data collection to ensure clarity, wording, logical sequence, and reliability of the tool. A slight modification was made to the final data collection tool based on the pretest. The data for the main study were not collected from PLHIV who participated during the pretest, and the collected data for the pretest were excluded from the final analysis.

### 2.5. Data Analysis Management

Data that passed through quality control were entered into Statistical Package for Social Science (SPSS) Version 25.0 statistical packages for Windows. A Kolmogorov–Smirnov statistical test was used to test the normality of the data, and the data was considered skewed when the *p* value is < 0.05. Normally distributed and skewed continuous variables were expressed by mean (±SD) and median with interquartile range (IQR), respectively. Categorical variables were summarized as frequency (percentage) of the total. Variables with a *p* value < 0.2 in the bivariate binary logistic regression analysis were included in the multivariable logistic regression. Adjusted odds ratio (AOR) with 95% CI was used to report the strength of association between the dependent and independent variables, and a *p* value < 0.05 was taken as statistically significant to declare the association. The goodness fit of the model was confirmed by the Hosmer and Lemeshow test (*p* value = 0.626).

## 3. Result

### 3.1. Sociodemographic Characteristics

#### 3.1.1. Sociodemographic Individual Factors, Physical, and Social Environment

Overall, 494 PLHIV agreed to take part, giving a response rate of 96.1%. The mean age of PLHIV was 41.85 ± 12.3 years, and more than half (64.6%) of them were females. Less than a quarter (20%) of PLHIV had no formal education. Over three-fourths of PLHIV were married (84.8%) and resided in an urban area (92.3%) (Table 1).

### 3.2. Condition-Specific Factors

The median (IQR) duration of the PLHIV since they were diagnosed with HIV and started ART was 144 (85) and 132 (84) months, respectively. More than three-fourths of PLHIV did not know their WHO HIV/AIDS stage (84.8%), did not have any additional medically diagnosed disease (83.2%), and used only one tablet per day for their HIV treatment (80.4%). Furthermore, sexual intercourse was the most common route of infection for the majority (53.4%) of PLHIV (Table 2).

### 3.3. Process of SM

In the process of SM, half (50.6%) and around one-fourth (26.1%) of PLHIV had poor self-efficacy and poor social support, respectively. Less than one-fourth of PLHIV were nonadherent to their ART medication (18.8%) and think the counseling they got during follow-up was inadequate (22.1%) (Table 3).

### 3.4. SM

**Figure 1:**
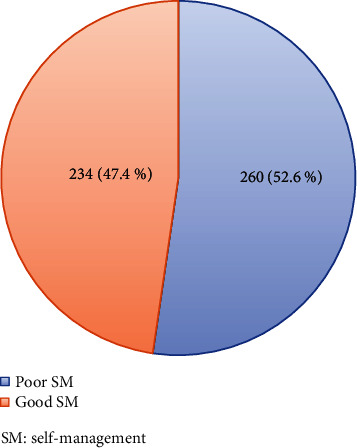
Self-management of people living with human immunodeficiency virus at the University of Gondar at the University of Gondar Comprehensive Specialized Hospital, antiretroviral clinic; May 20–July 30, 2022 (*N* = 494). SM, self-management.

Above half (52.6%, [95% CI: 48%–57%]) of PLHIV had poor SM (Figure 1). The median (IQR) SM score of PLHIV was 39 (9). The median (IQR) SM score of daily health practice, resource mobilization for HIV, and chronic nature of HIV were 25 (7), 0 (3), and 13 (2), respectively. PLHIV had a higher median (IQR) score on the chronic nature of the HIV domain than the two SM domains (Table 4).

### 3.5. Factors Affecting SM

Multivariable binary logistic regression shows that the odds of having poor SM were two times higher among unemployed PLHIV (AOR = 2.49, 95%CI = 1.19, 5.19, *p* = 0.015) than PLHIV who had governmental jobs. PLHIV living alone has two times higher odds of having poor SM (AOR = 2.16, 95%CI = 1.12, 4.17, *p* = 0.022) than PLHIV who lives with their family. The odds of having poor SM were three times higher among PLHIV with poor self-efficacy (AOR = 3.04, 95%CI = 1.83, 5.06, *p* < 0.001) than PLHIV with good self-efficacy. PLHIV who were unfamiliar with the management of HIV-related symptoms (AOR = 3.59, 95%CI = 2.08, 6.20, *p* < 0.001) had three times poorer SM than PLHIV who were familiar with the management of HIV-related symptoms.

There was a significant difference between the SM of PLHIV based on their social support (*p* < 0.001). The odds of having poor SM among PLHIV who had poor social support (AOR = 3.02, 95%CI = 1.54, 5.93, *p* = 0.001) were three times higher than those PLHIV who had strong social support. (Table 5).

## 4. Discussion

This study was aimed at assessing SM and its associated factors among PLHIV at the UOGCSH ART clinic. Out of the 494 PLHIV participants in this study, more than half (52.6%, 95% CI: 48%–57%) of them had poor SM. SM has three domains; from these domains, the highest median (IQR) scores (13 (2)) were recorded on the living with chronicity of HIV domain, and the least median (IQR) scores (0 (3)) were recorded on the social support domain. This result is consistent with studies conducted in Ethiopia [23], China [25], and Korea [24].

In this study, the overall SM of participants was inadequate, and it is consistent with studies conducted in Ethiopia [23], China [25, 26], and Korea [24]. However, the SM of PLHIV in the United States was moderate [10]. The difference might be because patients living in developing countries like Ethiopia have a lower health literacy level and a lack of SM interventional programs. This in turn decreases an individual's day-to-day assessment, understanding, evaluation, and use of health information, which plays a critical role in SM [34–37]. Since participant SM is inadequate, it is good to incorporate SM programs as usual patient care services for PLHIV.

In this study, PLHIV, who lived alone, had poor SM. This might be because PLHIV living alone has poor family support in every aspect of their living condition [38] and this leads to poor SM [25]. In reverse, IFSMT and the model of self-stigma and psychological well-being among PLHIV stated that because of better support in family and marriage, PLHIV living with family have good SM [39, 40]. Social support also greatly affects SM, especially in disease conditions where stigma is higher. In this study, PLHIV with both poor and intermediate social support had poor SM. This result was in line with the study conducted in China [25]. This may be in reverse; good social support is associated with better psychosocial activity [41], reduced complaints of disease management, and an improved attitude toward SM, which directly improves SM [38]. The government and community should promote stigma reduction and good social support to improve the SM of PLHIV.

PLHIV, who were unfamiliar with the management of HIV-related symptoms, have poor SM in this study. This might be due to the ability to self-regulate affecting self-efficacy and participation in SM [42–44]. In addition, the poor process of SM may create a worsening of SM and poor treatment outcomes [40]. Thus, incorporating IFSMT in the management protocol of PLHIV and giving emphasis on the management of HIV/AIDS-related symptoms can make a massive improvement in PLHIV's self-regulation ability. In addition, this study revealed that poor SM is higher among unemployed PLHIV than those who work in a governmental office. This may be because some components of SM may require income to execute them, which may not be affordable by unemployed PLHIV. So creating job opportunities for PLHIV can improve the lifestyle and SM of PLHIV.

PLHIV who have poor self-efficacy had poor SM in this study. This finding is supported by many previous studies [23, 40, 45–48]. This is maybe because self-efficacy is the heart of SM [31], and it is important as it influences the way patients follow healthcare worker's recommendations [49]. Self-efficacy has been consistently associated with HIV treatment adherence [50] and perceived social support [49], and it is a direct predictor of viral load [51]. For these reasons, it is considered an important and critical predictor of SM and health outcomes in PLHIV [50, 52]. Even though the specific component that improves SM is unclear, it is better to encourage PLHIV to practice a full scope of self-efficacy.

In a similar fashion to a study conducted in Debre Markos, Ethiopia [23], our study also finds that PLHIV who are not supported by adherence groups had poor SM. This may be because PLHIV who are supported by adherence support groups may have better economic and psychosocial support that reduces the burden of disease and makes them active participants in their disease management than PLHIV who are not supported by adherence support groups [53]. So including PLHIV in available adherence support groups or facilitating the establishment of new support groups can improve their SM.

The finding of this study helps ART clinic healthcare professionals in this study setting to intervene based on identified gaps and helps many social support groups to include SM skills in their education programs. In addition, the findings of this study will serve as a baseline and initiate researchers to further assess both proximal (cost associated with direct or indirect cost) and distal (clinical outcome and health status, quality of life, and well-being) outcomes of SM. In collaboration with other studies, especially interventional ones, this study will help policymakers and the Minister of Health to incorporate SM programs or educational programs that strengthen the SM of patients.

### 4.1. Limitations of the Study

Being a single-center study may limit its generalizability, and due to the intrinsic nature of a cross-sectional study, it cannot show the cause-and-effect relationship between the independent and dependent variables. This study also did not assess the proximal (cost associated with direct or indirect cost) and distal outcomes (clinical outcome and health status, quality of life, and well-being) of SM. Despite the limitations, this study was conducted with an adequate sample size, and an effort was made to test the association of multiple risk factors with SM.

## 5. Conclusion

More than half of PLHIV SM is poor in this study setting. Being unemployed, living alone, unable to manage HIV-related symptoms, having poor and intermediate social support, poor self-efficacy, and being unsupported by adherence support groups were significantly associated with poor SM. This study finding supports IFSMT and previously published literatures regarding factors that affect SM. To begin with, PLHIV can promote good SM by improving social support, self-regulation ability, and self-efficacy. In addition, adherence support groups should encourage and facilitate the participation of PLHIV in adherence support groups. Furthermore, the hospital should incorporate SM programs and SM skills development in the usual PLHIV care service. A future researcher may focus on the proximal (cost associated with direct or indirect cost) and distal outcome (clinical outcome and health status, quality of life, and well-being) of SM.

## Figures and Tables

**Table 1:** Sociodemographic individual factors and physical and social environment factors of people living with human immunodeficiency virus at the University of Gondar Comprehensive Specialized Hospital, antiretroviral clinic; May 20–July 30, 2022 (*N* = 494).

**Variable**	**Frequency**	**Percent**
Age		
≤ 41.85	257	52
> 41.85	237	48
Sex		
Male	175	35.4
Female	319	64.6
Education level		
No formal education	99	20
Formal education	395	80
Job		
Governmental job	87	17.6
Private organization	78	15.8
Farmer	18	3.6
Merchant	103	20.9
Student	31	6.2
Unemployed	149	30.2
Daily laborer	28	5.7
Living condition		
Live alone	118	23.9
Live with family	376	76.1
Marital status		
Single	75	15.2
Married	419	84.8
Residency		
Rural	38	7.7
Urban	456	92.3

**Table 2:** Sociodemographic condition-specific factors of people living with human immunodeficiency virus at the University of Gondar Comprehensive Specialized Hospital, antiretroviral clinic; May 20–July 30, 2022 (*N* = 494).

**Variable**	**Frequency**	**Percent**
When were you diagnosed as HIV positive (in a month)?		
Median (IOR) = 144 (85)		
For how long have you been on antiretroviral therapy (in months)?		
Median (IOR) = 132 (84)		
Do you know your WHO HIV stage?		
Yes	75	15.2
No	419	84.8
Has your treatment regimen changed?		
Yes	389	78.7
No	105	21.3
Did you have any additional medically diagnosed diseases?		
Yes	83	16.8
No	411	83.2
How many tablets are you taking for your HIV treatment?		
One	397	80.4
Two	74	15.0
Three	23	4.6
Have you ever experienced drug side effects?		
Yes	50	10.1
No	444	89.9
How did you acquire HIV?		
Sexual intercourse	264	53.4
MTCT	31	6.3
Accidentally by sharp material	51	10.3
I do not remember	148	30
Do you know your viral load?		
Yes	116	23.5
No	366	74.1
I do not know	12	2.4

Abbreviations: HIV, human immunodeficiency virus; IQR, interquartile range; MTCT, mother-to-child transmission; WHO, World Health Organization.

**Table 3:** Process of self-management of people living with human immunodeficiency virus at the University of Gondar Comprehensive Specialized Hospital, antiretroviral clinic; May 20–July 30, 2022 (*N* = 494).

**Variable**	**Frequency**	**Percentile**
Self-efficacy		
Poor self-efficacy	250	50.6
Good self-efficacy	244	49.4
I try to have a plan for SM of emotional distress		
Yes	280	56.7
No	214	43.3
I am familiar with the management of HIV-related symptoms		
Yes	194	39.3
No	300	60.7
Have you set a goal in the process of your HIV therapy?		
Yes	313	63.4
No	181	36.6
Social support		
Poor support	129	26.1
Intermediate support	181	36.7
Strong support	184	37.2
Adherence		
Adherent	401	81.2
Nonadherent	93	18.8
Did you support from an adherence support group?		
Yes	105	21.3
No	389	78.7
The counseling you got was adequate for the next HIV treatment		
Yes	385	77.9
No	109	22.1
Have you been encouraged to disclose your HIV status?		
Yes	328	66.4
No	166	33.6

Abbreviations: HIV, human immunodeficiency virus; SM, self-management.

**Table 4:** Self-management median (interquartile range) score of people living with human immunodeficiency virus at the University of Gondar Comprehensive Specialized Hospital, antiretroviral clinic; May 20–July 30, 2022 (*N* = 494).

**Variables**	**Median (interquartile range)**
Total SM score value (*N* = 60)	39 (9)
Domain 1: Daily health practice (*n* = 36)	25 (7)
Domain 2: Resource mobilization for HIV (*n* = 9)	0 (3)
Domain 3: Chronic nature of HIV (*n* = 15)	13 (2)

Abbreviations: HIV, human immunodeficiency virus; IQR, interquartile range; *N*, total score self-management; *n*, total score of each domain; SM, self-management.

**Table 5:** Factors that affect self-management of people living with human immunodeficiency virus at the University of Gondar Comprehensive Specialized Hospital, antiretroviral clinic; May 20–July 30, 2022 (*N* = 494).

**Variables**	**Poor SM**	**Good SM**	**COR (95% CI)**	**AOR (95% CI)** ^a^	**p** ** value**
Education level					
No formal education	67	32	2.19 (1.38, 3.49)	1.49 (0.74, 2.99)	0.268
Formal education	193	202	1.00	1.00	
Job					
Private organization	38	40	1.17 (0.63, 2.16)	1.50 (0.68, 3.29)	0.081
Farmer	12	6	2.46 (0.85, 7.16)	2.21 (0.45, 10.90)	0.311
Merchant	44	59	0.92 (0.52, 1.63)	0.93 (0.43, 2.01)	0.329
Student	13	18	0.89 (0.39, 2.04)	2.95 (0.73, 11.96)	0.861
Unemployed	95	54	2.15 (1.26, 3.71)	2.49 (1.19, 5.19)	0.130
Daily laborer	19	9	2.60 (1.06, 6.38)	2.53 (0.68, 9.45)	0.015⁣^∗^
Governmental job	39	48	1.00	1.00	0.168
Living condition					
Living alone	79	39	2.18 (1.41, 3.37)	2.16 (1.12, 4.17)	0.022⁣^∗^
Living with family	181	195	1.00	1.00	
Marital status					
Single	170	134	1.41 (0.98, 2.03)	1.08 (0.61, 1.92)	0.783
Married	90	100	1.00	1.00	
Residency					
Rural	25	13	1.81 (0.90, 3.62)	1.58 (0.61, 4.14)	0.349
Urban	235	221	1.00	1.00	
How many years since you were diagnosed?			0.997 (0.994, 1.000	0.999 (0.995, 1.00)	0.665
Do you know your HIV stage?					
Yes	28	47	1.00	1.00	
No	232	187	2.08 (1.26, 3.45)	1.226 (0.58, 2.60)	0.595
Is treatment changed?					
Yes	193	196	1.00	1.00	
No	67	38	1.79 (1.15, 2.79)	1.50 (0.79, 2.83)	0.213
Transmission route					
MTCT	10	21	0.35 (0.16, 0.77)	0.60 (0.15, 2.36)	0.263
Accidentally by sharp material	18	33	0.40 (0.22, 0.75)	0.44 (0.20, 1.01)	0.468
I do not remember	80	68	0.87 (0.58, 1.30)	0.91 (0.52, 1.59)	0.052
Sexual intercourse	152	112	1.00	1.00	0.728
Do you know your viral load?					0.340
Yes	46	70	1.00	1.00	
No	208	158	2.00 (1.31, 3.07)	1.53 (0.85, 2.75)	0.153
I do not know	6	6	1.52 (0.46, 5.01)	1.03 (0.20, 5.40)	0.971
Self-efficacy					
Poor self-efficacy	168	82	3.39 (2.34, 4.90)	3.04 (1.83–5.06)	< 0.001⁣^∗^
Good self-efficacy	92	152	1.00	1.00	
I try to have a plan for SM of emotional distress					
Yes	123	157	1.00	1.00	
No	137	77	2.27 (1.58, 3.27)	0.90 (0.53, 1.52)	0.686
I am familiar with the management of HIV-related symptoms					
Yes	69	125	1.00	1.00	
No	191	109	3.17 (2.18, 4.63)	3.59 (2.08, 6.20)	< 0.001⁣^∗^
Have you set a goal in the process of your HIV therapy?					
Yes	142	171	1.00	1.00	
No	118	63	2.26 (1.55, 3.29)	1.66 (0.99, 2.80)	0.055
Social support					
Poor support	94	35	4.80 (2.94, 7.85)	3.02 (1.54, 5.93)	< 0.001
Intermediate support	100	81	2.21 (1.45, 3.36)	2.71 (1.56, 4.720)	0.001⁣^∗^
Strong support	66	118	1.00	1.00	< 0.001⁣^∗^
Medication adherence					
Nonadherent	61	32	1.94 (1.21, 3.10)	1.80 (0.92, 3.49)	0.084
Adherent	199	202	1.00	1.00	
Did you support by adherence support group?					
Yes	19	86	1.00	1.00	
No	241	148	7.37 (4.31, 12.62)	17.17 (8.37, 35.22)	< 0.001⁣^∗^
The counseling you got was adequate for the next HIV treatment					
Yes	185	200	1.00	1.00	
No	75	34	2.39 (1.52, 3.75)	1.69 (0.91, 3.13)	0.096
Have you been encouraged to disclose your HIV status ?					
Yes	155	173	1.00	1.00	
No	105	61	1.92 (1.32, 2.82)	1.07 (0.62, 1.87)	0.800

Abbreviations: AOR, adjusted odds ratio; COR, crude odds ratio; HIV, human immunodeficiency virus; MTCT, mother-to-child transmission; SM, Self-management.

^a^Binary logistic regression analysis (AOR) with 95% CI was used to report the strength of the association.

⁣^∗^Statistically significant (*p* value < 0.05).

## Data Availability

The datasets used and/or analyzed during the current study are available from the corresponding author upon reasonable request.

## Nomenclature

$: dollar
ART: antiretroviral therapy
CD4: cluster of differentiation 4
HAART: highly active antiretroviral therapy
HIV/AIDS: human immunodeficiency virus/acquired immune deficiency syndrome
IFSMT: individual family self-management theory
IQR: interquartile range
PLHIV: people living with human immunodeficiency virus
SM: self-management
UOGCSH: University of Gondar Comprehensive Specialized Hospital
USA: United States of America
